# The predictive value of serum neurofilament light chain in achieving no evidence of disease activity-3 (NEDA-3) status in treatment-naïve patients with multiple sclerosis

**DOI:** 10.1007/s00415-026-13709-0

**Published:** 2026-03-09

**Authors:** Evangelia Makrina Dimitriadou, Dimitrios Tzanetakos, Aikaterini Theodorou, Alexandra Akrivaki, Dimitrios Papadopoulos, Vasileios Mastorodemos, Aleksandra Maleska, Stefania Alexia Sotirli, Nikolaos Fakas, Christos Stergiou, Dimitrios Kitsos, Vasileios Giannopapas, Christos Moschovos, Sotirios Giannopoulos, Jens Kuhle, Georgios Tsivgoulis, John S. Tzartos

**Affiliations:** 1 Second Department of Neurology, National and Kapodistrian University of Athens, “Attikon” University Hospital, Rimini 1, 12462 Athens, Greece; 2 Medical School, European University of Cyprus, Nicosia, Cyprus; 3 Neurology Department, University Hospital of Heraklion, Heraklion, Greece; 4 Department of Neurology, University Hospital and University of Basel, Basel, Switzerland; 5 Department of Neurology, Metropolitan General Hospital, Athens, Greece; 6 Department of Neurology, 401 General Military Hospital, 11525 Athens, Greece; 7 Tzartos NeuroDiagnostics, Athens, Greece

**Keywords:** Serum neurofilament light chain, Multiple sclerosis, Predictive value, Biomarker, NEDA-3

## Abstract

**Background:**

Serum neurofilament light chain (sNfL) has emerged as a biomarker reflecting neuroaxonal damage. The role of sNfL in predicting clinical outcome and guiding therapeutic management in multiple sclerosis (MS) is an ongoing research topic. We sought to evaluate sNfL utility for the prediction of achieving No Evidence of Disease Activity-3 (NEDA-3) among MS patients.

**Methods:**

We conducted a prospective cohort study including MS patients with a single sNfL measurement at first clinical evaluation. Follow-up was performed every 6 months/clinical relapse and included clinical and MRI evaluation. The main outcome was NEDA-3 achievement during follow-up. sNfL levels were measured, using single-molecule-array (SIMOA) assay. sNfL age- and BMI-normalized Z-scores > 1.5 were defined as high. Linear regression analyses were performed to identify predictors of baseline sNfL Z-score and multivariable Cox proportional hazard regression models were used to evaluate predefined outcomes.

**Results:**

We included 125 patients [age at sNfL measurement:40 ± 12, 30% men, 82% relapsing–remitting and 18% progressive MS]:37 treatment-naïve patients, 88 patients under Disease-Modifying-Treatment (DMT) with 19.1 (Interquartile Range:12.1–24.5) months median follow-up. Prior DMT use [β:-0.93; 95%CI:-1.48– -0.38 *p*-value:0.001] was negatively associated with baseline sNfL Z-scores. Gadolinium-enhancing lesions were associated with higher sNfL Z-scores [adjusted standardized linear regression coefficient-β:0.68; 95%CI:0.03–1.32; *p*-value:0.036], contrary to T2-weighted lesion activity (β:0.40; 95%CI:0.17–0.94; *p*-value:0.232). In treatment-naive patients, high sNfL Z-scores were associated with lower probability of achieving NEDA-3 during follow-up [hazard-ratio:0.35; 95%CI:0.13–0.96; *p*-value:0.041].

**Conclusions:**

This study demonstrated that gadolinium-enhancing lesions, indicative of active inflammation, are associated with elevated sNfL Z-scores, contrary to T2-weighted lesion-activity and highlights the prognostic value of sNfL in achieving NEDA-3 in treatment-naive patients.

## Introduction

In Multiple Sclerosis (MS), the identification of easily accessible biomarkers remains a key objective in ongoing research. While Magnetic Resonance Imaging (MRI) has been considered as the laboratory gold standard for detecting disease activity, either through contrast-enhancing lesions or new/enlarging T2-weighted lesions, serum neurofilament light chain (sNfL) has emerged as a promising biomarker reflecting neuroaxonal damage and potentially serving as a prognostic biomarker of disease progression [1, 2].

There is substantial evidence supporting the association between sNfL levels, disease activity, and their prognostic significance [3]. More specifically, high sNfL levels have been associated with future clinical activity, MRI activity, Expanded Disability Status Scale (EDSS) worsening and brain atrophy [4–7]. Nevertheless, the role of sNfL in the pathogenetic mechanism of MS and its integration as a biomarker in routine clinical practice, remains to be fully elucidated and further investigation is required in order to establish the utility and positioning of sNfL as a biomarker in the evidence based monitoring and management of the disease.

In view of the former considerations, we conducted an observational prospective cohort study, aiming to evaluate the value of baseline sNfL levels, in predicting the achievement of No Evidence of Disease Activity 3 (NEDA-3) status by measuring sNfL during the first clinical evaluation in our MS outpatient clinic; baseline sNfL levels were measured either at the time of diagnosis or during the disease course.

### Materials and methods

We prospectively evaluated real world data of MS patients with available sNfL measurements at first clinical evaluation, during a two-year period (June 2023– Apr 2025) in our Outpatient Service (Second Department of Neurology, National and Kapodistrian University of Athens, “Attikon” University Hospital). This study was conducted in accordance with the Declaration of Helsinki principles. Institutional review board approval was obtained from the Ethics Committee of “Attikon” University Hospital (decision number: ΕΒD 318/29- 04–2024). The study was also performed in accordance with the Strengthening the Reporting of Observational Studies in Epidemiology (STROBE) guidelines for reporting observational research [8]. All participants or their legal representatives provided signed informed consent.

The inclusion criteria were (a) age ≥ 18 years old, (b) diagnosis of MS according to the McDonald Criteria 2017, (c) availability of sNfL measurements at baseline evaluation, and (d) availability of imaging data including brain, cervical and thoracic spinal cord MRI, annually or close to a clinical relapse. Patients were evaluated prospectively using the EDSS and NEDA-3 assessment every 6 months or sooner in case of a relapse [9, 10]. MRI and EDSS assessments were performed according to a predefined follow-up schedule, aligned with routine clinical practice. When minor deviations occurred, the closest available assessment was used. Participants with incomplete baseline or follow-up assessments, or who did not consent, were excluded.

The following data were collected for each patient: age, sex, disease duration from onset to baseline sNfL measurement, treatment, Expanded Disability Status Scale (EDSS) and clinical and/or Magnetic Resonance Imaging (MRI) activity, a single baseline sNfL measurement at first clinical evaluation, months until last follow-up assessment, No Evidence of Disease Activity-3 (NEDA-3) and EDSS assessment, and radiological findings during last follow-up. Every follow-up assessment included clinical and MRI evaluation.

Measurements of sNfL levels were performed during the intial clinical evaluation in our outpatient service (Second Department of Neurology, National and Kapodistrian University of Athens, “Attikon” University Hospital). A peripheral blood sample was drawn from each patient during the baseline clinical evaluation. Single-molecule-array (SIMOA) assay was used for the measurement of sNfL levels. The Nf-Light Advantage PLUS kit of Quanterix was used. sNfL Z‑scores, adjusted for age and BMI, represent a validated prognostic biomarker in MS. High sNfL Z-scores were defined as values above 1.5, whereas values less or equal to 1.5 were defined as low [11, 12]. The threshold > 1.5 is supported by large cohort evidence as indicating higher risk for future clinical or MRI disease activity. The sNfL Z-score > 1.5 was associated with greater risk of future clinical or MRI activity (OR: 3.15; 95% CI, 2.35—4.23; *P* < 0.0001); this was also the case in patients in NEDA status (OR: 2.66; 95% CI, 1.08—6.55; *P* = 0.034) [6, 12, 13].

All brain, cervical and thoracic spinal cord MRI results were evaluated by two independent investigators (E-M.D and D.T.) who were blinded to clinical characteristics. All patients underwent either a 1.5 Tesla Tesla (Philips Healthcare, Best, The Netherlands) or a 3 Tesla MRI scan Siemens Magnetom Prisma, Siemens Healthineers, Erlangen, Germany) and continued to be scanned in the same MRI magnetic field strength on follow-up. A comprehensive brain, cervical and thoracic spinal cord MRI protocol with multiplanar reconstruction was used in all participants, which included the following sequences: the T2-weighted sequence, the 3D fluid-attenuated inversion recovery (FLAIR) sequence, the diffusion-weighted imaging (DWI) sequence, the susceptibility-weighted imaging (SWI) sequence and the post-contrast T1-weighted sequence for the brain MRI, as well as T1-weighted, T2-weighted, short tau inversion recovery (STIR), and post-contrast T1-weighted sequences for the spinal cord MRI.

Clinical activity at baseline was defined as clinical relapse occurring within 30 days prior to the baseline evaluation and MRI activity was defined as gadolinium-enhancing lesions or new or enlarging T2 lesions within 30 days prior to the baseline evaluation.

Achievement of NEDA-3 during follow-up was predefined as the main outcome of our study. EDSS was used as a clinician-administered assessment scale of worldwide acceptance in order to evaluate the functional systems of the central nervous system [9] Moreover, NEDA-3, defined as a composite of three related measures, namely no clinical relapses, no sustained disability progression (as defined by no increase in the EDSS-score), and no activity seen on MRI (new or enlarging T2 hyperintense lesions or gadolinium-enhancing lesions) during a specified time period was used [10]. Achieving NEDA-3 during the last follow-up was defined as the main outcome.

### Statistical analysis

Categorical variables are presented as mean ± standard deviations (SD) for continuous variables with normal distribution or as median with interquartile ranges (IQRs) in cases of skewed distributions. Comparisons of different variables were expressed using the unpaired t-test or the Mann–Whitney U test. Statistical significance was defined as a *p*-value < 0.05.

Single and multivariable linear regression models were performed to identify predictors of baseline sNfL Z-score. Univariable and multivariable Cox proportional hazards regression models were used to evaluate the association of sNfL with NEDA-3 during last follow-up. The model included baseline sNfL levels (high vs. low), EDSS score at the time of baseline sNfL measurement, MS subtype at baseline (relapsing–remitting MS vs. progressive MS), and the presence of gadolinium-enhancing (Gd +) lesions at baseline. Covariates were selected a priori based on clinical relevance and prior literature, and no automated variable selection procedures were applied. The proportional hazard assumption was assessed using Schoenfeld residuals and log–log survival plots. All analyses were performed using the R–software version 2025.05.0 + 496 and GraphPad Prism-10.

## Results

**Fig. 1:**
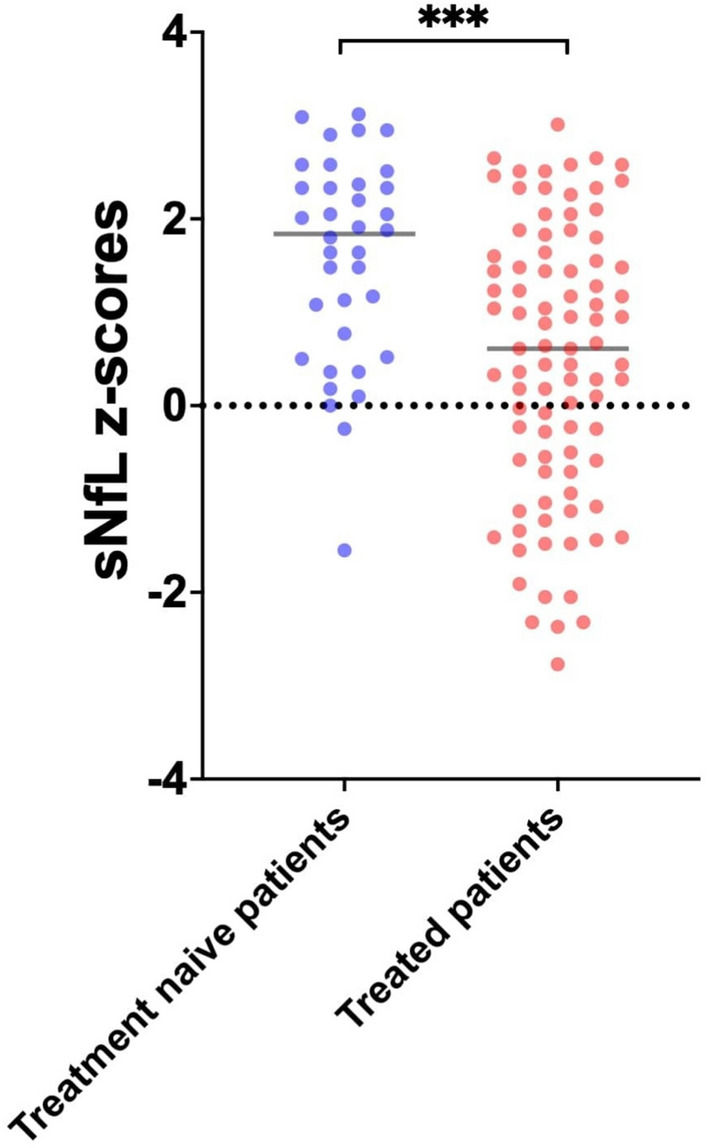
sNfL Z-scores among treatment naïve patients and patients treated with any disease modifying treatment at baseline

Our cohort comprised 125 MS patients [age at sNfL measurement: 40 ± 12, 38 (30%) men, 103 (82%) with relapsing–remitting MS (RRMS) and 22 (18%) with progressive MS (PMS)]. Thirty-seven patients (30%) were treatment-naive at baseline sNfL measurement, whereas 88 patients (70%) were under disease modifying treatments (DMTs). Baseline characteristics, treatment and neuroimaging data from the baseline and last follow-up evaluation are summarized in the Table 1**.** A relapse within 30 days from the measurement was reported in 40 (32%) patients, whereas gadolinium-enhancing activity close to baseline sNfL measurement was detected in 23 (18%) patients. Previous treatment with any DMT was associated with significantly lower sNfL Z-score at baseline (Fig. 1; *p*-value < 0.001). The median follow-up period in our cohort was 19 (IQR: 12–25) months.

**Table 1:** Baseline characteristics and follow-up evidence

Variables	Total (N = 125)
Baseline characteristics and data from last follow-up	
Age at baseline sNfL measurement, Mean (SD)	39.8 (11.8)
Sex – Male, n (%)	38 (30%)
Disease duration in years (from onset to baseline sNfL measurement), Median—IQR	5.5 (1.3 – 11.5)
MS type (at the time of 1^st^sNfL measurement), n (%)	
RRMS	103 (82%)
PMS	22 (18%)
Therapy at the time of baseline sNfL measurement, n (%)	
No DMT	37 (30%)
Any DMT*	88 (70%)
EDSS at the time of baseline sNfL measurement, n (%)	
EDSS ≤ 4	110 (88%)
EDSS > 4	15 (12%)
Clinical MS activity close to baseline sNfL measurement, n (%)	
No	85 (68%)
Yes	40 (32%)
Gd + activity close to baseline sNfL measurement, n (%)	
No	102 (82%)
Yes	23 (18%)
T2 activity close to baseline sNfL measurement, n (%)	
No	108 (86%)
Yes	25 (20%)
Time elapsed between baseline MRI and sNfL measurement (days), Median – IQR	10 (6–14)
Baseline sNfL measurement (pg/ml), Median – IQR	8.9 (6.2 – 14.3)
Z-score of baseline sNfL measurement, Mean (SD)	0.78 (1.45)
Baseline sNfL value, n (%)	
High (sNfL Z-score > 1.5)	45 (36%)
Low (sNfL Z-score ≤ 1.5)	80 (64%)
Last follow-up evaluation	
Months between baseline sNfL measurement and last follow-up, Mean (SD)	19.1 (8.2)
NEDA-3 during last follow-up evaluation, n (%)	
0	53(42%)
1	72 (58%)
EDSS worsening during last follow-up, n (%)	
No	120 (96%)
Yes	5 (4%)
MRI activity during last follow-up, n (%)	
No	86 (69%)
Yes	39 (31%)
Clinical activity during last follow-up, n (%)	
No	106 (85%)
Yes	19 (15%)

EDSS: Expanded Disability Status Scale, Gd + : Gadolinium enhancement, IQR: Interquartile Range, MRI: Magnetic Resonance Imaging, MS: Multiple Sclerosis, NEDA-3:No Evidence of Disease Activity 3, PMS: Progressive Multiple Sclerosis, RRMS: Relapsing Remitting Multiple Sclerosis, SD: Standard Deviation, sNfL: serum neurofilament light chain * Patients receiving any of the following DMTs between DMT initiation Α. Platform therapies: beta interferons, glatiramer acetate. B. Oral therapies: teriflunomide, dimethyl fumarate, fingolimod, ozanimod, siponimod, cladribine, azathioprine, mycophenolate mofetil. C. Monoclonal antibodies: natalizumab, alemtuzumab, ocrelizumab, ofatumumab, rituximab

**Fig. 2:**
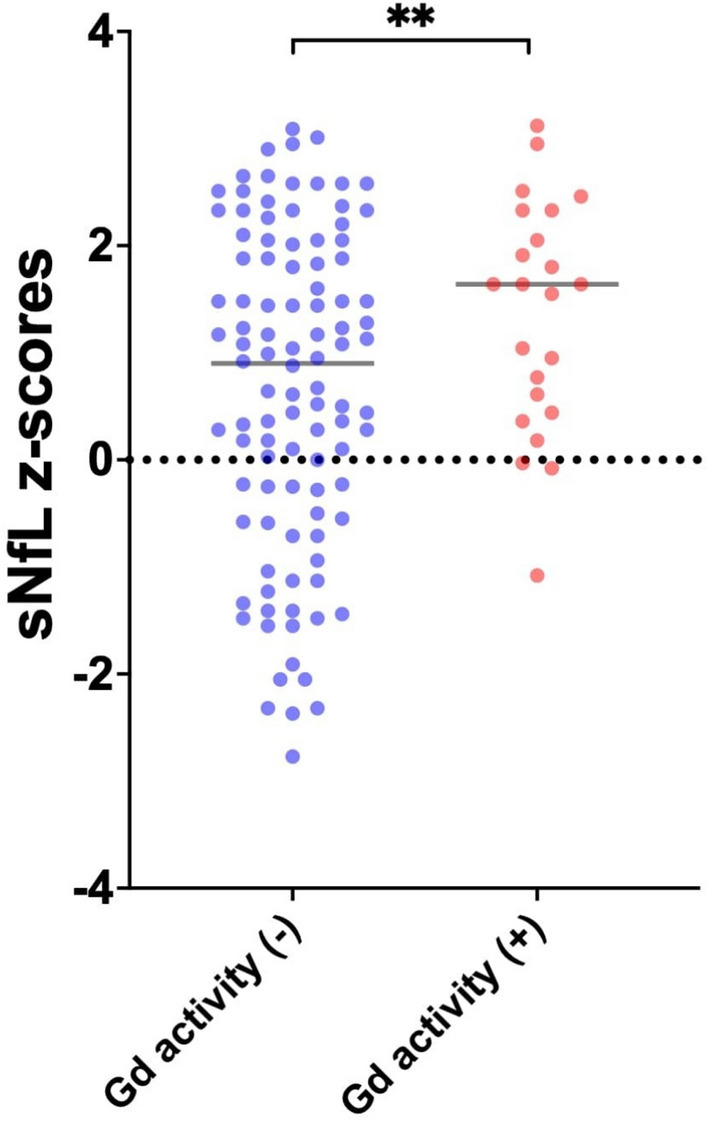
sNfL Z-scores among patient with or without gadolinium activity at baseline

Results from linear regression analysis, assessing the predictors of baseline sNfL Z-score are summarized in Table 2. In multivariable regression models, a negative trend towards disease duration prior to the baseline sNfL measurement and baseline sNfL Z-score was observed [adjusted standardized linear regression coefficient (beta)—β: −0.03; 95%CI: −0.05– −0.01; *p*-value: 0.091]. Moreover, any prior DMT [β: −0.93; 95%CI: −1.48– −0.38; *p*-value: 0.001] was negatively associated with baseline sNfL Z-score. Interestingly, MRI gadolinium-enhancing lesions detected close to sNfL measurement (within 30 days from the measurement) were independently associated with higher sNfL Z-scores [β: 0.68; 95%CI: 0.03–1.32; *p*-value: 0.036; Fig. 2], in contrast to depicting T2-weighted-lesion activity (β: 0.40; 95%CI: −0.17 –1.04; *p*-value: 0.232).

**Table 2:** Simple and Multiple linear regression models to evaluate the predictors of baseline sNfL levels, calculated at Z-scores

	Simple linear regression models				Multiple linear regression model	
Covariate/factor	Unadjusted standardized linear regression coefficient	Lower 95% CI	Upper 95% CI	p-value	Adjusted standardized linear regression coefficient	p-value
Age at MS onset, per 1-year increase	− 0.020	− 0.043	0.002	0.082		
Male (Sex)	0.426	− 0.129	0.982	0.131		
Disease duration prior to baseline sNfL, per 1-year increase	− 0.041	− 0.074	− 0.008	0.013	− 0.027	0.091
MS type at the time of baseline sNfL (RRMS vs. PMS)	− 0.061	− 0.738	0.616	0.859		
EDSS at the time of baseline sNfL	− 0.039	− 0.192	0.114	0.613		
Treatment vs no treatment at the time of baseline sNfL	− 1.115	− 1.646	− 0.577	< 0.001	− 0.931	0.001
Gd + activity at baseline	0.701	0.047	1.355	0.036	0.675	0.036
T2 activity at baseline	0.401	− 0.261	1.063	0.232		
Clinical activity at baseline	0.382	− 0.174	0.938	0.176		

CI: Confidence Interval, EDSS: Expanded Disability Status Scale, Gd + : Gadolinium enhancement, MS: Multiple Sclerosis, PMS: Progressive MS, RRMS: Relapsing remitting MS, sNfL: Serum neurofilament light chain

**Fig. 3:**
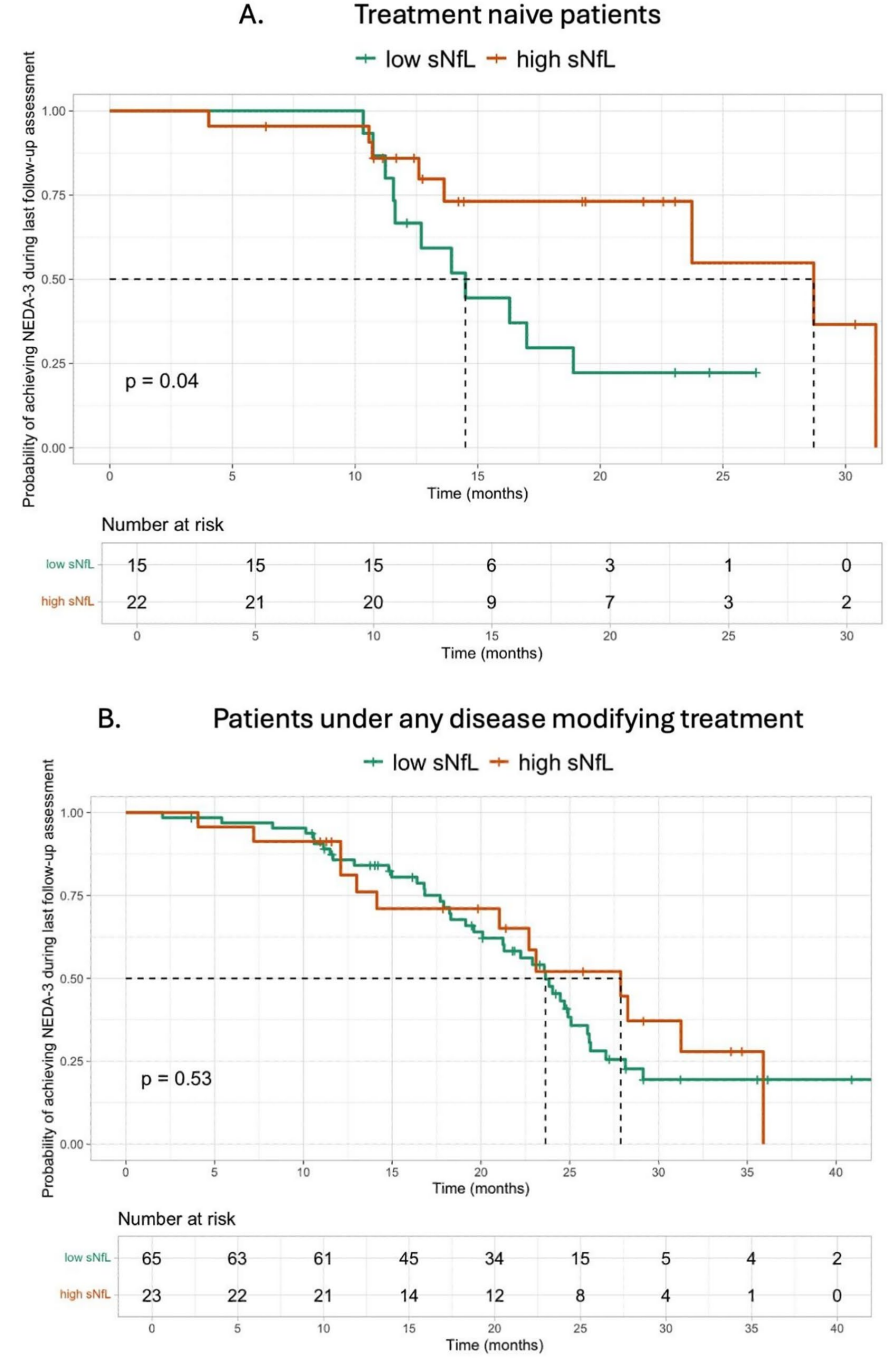
Kaplan–Meier Curves of Risk of achieving NEDA-3 at last follow-up by Serum Neurofilament Light Chain (sNfL) Z-scores stratified by treatment naïve patients (Panel A) vs. treated patients under any treatment (Panel B)

Moreover, higher sNfL Z-scores were independently associated with a lower probability of achieving NEDA-3 at the last follow-up assessment, among treatment-naive patients [hazard ratio (HR): 0.35; 95%CI:0.13–0.96; *p*-value = 0.041; Fig. 3]. Among 22 treatment-naïve patients with high sNfL Z-scores 14 did not achieve NEDA-3 status due to EDSS worsening in 2 patients, clinical relapse in 1 patient, MRI activity in 6 patients, clinical relapse and MRI activity in 5 patients. Nevertheless, this association failed to reach statistical significance among patients treated with any DMT (Fig. 3; Table 3). NEDA-3 achievement tended to be more frequent in patients with low sNfL Z-scores compared to the rest (64% vs. 47%; *p*-value: 0.064).

**Table 3:** Multivariable Cox Regression Models to Test Associations Between sNfL Levels and the Risk of achieving NEDA-3 during the last follow-up among treatment naïve patients vs. patients at any treatment

	Achieving NEDA-3 at the last follow-up			
	Treatment naïve patients (n = 37)		Patients at any treatment (n = 88)	
	HR (95% CI)	p-value	HR (95% CI)	p-value
High vs low baseline sNfL levels	0.35 (0.13 – 0.96)	0.041	1.02 (0.53 – 1.97)	0.942
EDSS at the time of baseline sNfL	1.25 (0.67 – 2.37)	0.469	0.91 (0.76 – 1.09)	0.326
MS type at the time of baseline sNfL (RRMS vs. PMS)	0.52 (0.04 – 7.01)	0.620	0.68 (0.30 – 1.53)	0.353
Gd + activity at baseline	1.00 (0.96 – 1.05)	0.926	0.07 (0.01 – 0.48)	0.007

CI: Confidence Interval, EDSS: Expanded Disability Status Scale, Gd + : Gadolinium enhancement, HR: Hazard Ratio, MS: Multiple Sclerosis, NEDA-3: No Evidence of Disease Activity 3, PMS: Progressive MS, RRMS: Relapsing remitting MS, sNfL: serum neurofilament light chain

## Discussion

This study demonstrated a statistically significant association between gadolinium-enhancing lesions and baseline elevated sNfL Z-scores. Moreover, the primary outcome of the study, which was the evaluation of the prognostic value of sNfL in predicting the achievement of NEDA-3 status, was confirmed in treatment-naive patients.

The association between gadolinium-enhancing lesions on MRI and elevated baseline sNfL Z-scores, is consistent with existing evidence demonstrating a positive correlation between gadolinium enhancing lesions and sNfL levels [14]. Gadolinium-enhancing lesions indicate blood–brain barrier disruption and active inflammation, while sNfL elevation indicates neuroaxonal damage [14]. In our cohort, sNfL levels were measured relatively close to the time of brain MRI acquisition, supporting the observed association between elevated sNfL and Gd + activity, and the lack of correlation with T2 lesion activity. The absence of a similar association between sNfL Z-scores and T2-weighted lesion activity may be due to the fact that gadolinium enhancing lesions, which represent active inflammation and have a greater impact on the extent of increment of sNfL levels compared to T2-weighted MRI lesions, with absence of gadolinium enhancement that primarily reflect chronic tissue changes and cumulative disease burden rather than ongoing inflammatory activity and acute neuronal damage, which are directly reflected by sNfL [14].

The negative statistical association between baseline sNfL Z-scores and disease duration in the treated subgroup of our cohort, may reflect the effect of DMTs on MS pathophysiology and natural course of the disease. Previous studies have demonstrated association between DMTs and lower sNfL Z-scores, with DMT exposure been correlated with reduction in sNfL levels [2, 3, 15, 16]. This is further supported by the negative association between the sNfL Z-scores and DMTs. observed also in the present study.

Regarding the primary outcome of this study, which was the prognostic value of baseline sNfL Z-score in relation to NEDA-3 status, a clear association was observed in treatment-naïve patients. Specifically, treatment-naïve patients with high baseline sNfL Z-score were less likely to achieve NEDA-3 status during follow-up period (HR:0.35, 95%CI:0.13 – 0.96). This aligns with existing evidence indicating that elevated baseline sNFL levels are associated with increased disease activity and disability in treatment-naive MS patients [16]. Specifically, treatment-naïve patients with high sNfL Z-scores exhibited a statistically significant lower probability of achieving NEDA-3. This supports the concept of using sNfL Z-scores for the prediction of EDSS worsening and disease relapses [6]. The fact that there was no statistical significance between sNfL Z-scores and achieving NEDA-3 status in patients under any DMT, may reflect the positive effect of DMTs on both disease activity, by minimizing disease worsening and relapses and thus sNfL Z-score [2, 3, 14, 15]. Longer follow-up periods are required in order to ensure more accurate conclusions about the prognostication of sNfL Z-scores in this group of patients. Therefore, in treatment-naïve patients, baseline sNfL Z-scores might have a greater utility in disease prognosis and in therapeutic decision making at the onset of the MS course***.*** Despite being derived from small subgroups, these findings support the hypothesis that sNfL may provide complementary prognostic information for EDSS worsening and disease relapses at treatment initiation and not a standalone decision-making tool [6]. When considered alongside established predictors such as baseline EDSS, MRI inflammatory activity, and MS subtype, sNfL may provide additional biological insight into disease aggressiveness and help identify patients who may benefit from earlier initiation of higher-efficacy DMTs. Importantly, this approach would complement, rather than replace, existing clinical and radiological frameworks. Integration of sNfL with current predictive tools aligns with emerging models of personalized MS management, although prospective studies with larger treatment-naïve cohorts are needed to validate thresholds, assess incremental predictive value, and determine whether sNfL-guided treatment strategies improve long-term outcomes.

Our study has several limitations, including first the moderate sample size, which may decrease statistical power and limit the robustness of subgroup analyses, and second the mean follow-up duration of 19.1 months, limiting access to long-term data on disease activity and progression. Although follow-up duration differed among patients, NEDA-3 was assessed within comparable observation periods. This variability could potentially introduce detection bias; however, the use of standardized outcome definitions likely minimized its impact on the observed associations. Third, our cohort was recruited from a tertiary outpatient clinic, which may introduce selection bias and limit generalizability to broader MS populations. Fourth, MRI scans were acquired using both 1.5 T and 3 T scanners, introducing heterogeneity in image quality and lesion detection. Moreover, the discrepancies between treatment-naïve patients and patients under any efficacy treatment at baseline and between relapsing remitting MS and progressive MS patients should also be acknowledged. Although PMS patients comprised a minority of the cohort, their distinct inflammatory profiles and sNfL dynamics could have influenced the observed associations. Notably, inclusion of MS subtype as a covariate in the multivariable models of this study did not yield statistically significant effects. The use of DMTs during follow-up was not included in the statistical models, as all patients were receiving treatment and the limited sample size precluded analyses comparing different DMTs. This constrains the assessment of whether follow-up treatment status modifies the relationship between sNfL and clinical outcomes and may limit the the general applicability and clinical relevance of our findings. Finally, the relatively small number of patients achieving NEDA-3 during last follow-up limited the number of covariates that could be included in multivariable models, and although clinically relevant variables were selected a priori, residual overfitting cannot be excluded. These implications could be eliminated and further evaluated in future larger prospective studies.

In conclusion, our findings suggest that baseline sNfL Z-score in treatment-naïve MS patients may be a valuable prognostic biomarker for future disease activity, which may guide disease stratification and therapeutic strategies. However, further studies with larger datasets are warranted to validate and extend these findings.

## Data Availability

All data needed to evaluate the conclusions in the paper are present in the manuscript. Additional data related to this paper may be obtained from the corresponding author, upon reasonable request.
